# A qualitative analysis of vaccine decision makers’ conceptualization and fostering of ‘community engagement’ in India

**DOI:** 10.1186/s12939-020-01290-5

**Published:** 2020-10-20

**Authors:** Tapati Dutta, Beth E. Meyerson, Jon Agley, Priscilla A. Barnes, Catherine Sherwood-Laughlin, Jill Nicholson-Crotty

**Affiliations:** 1 Room No. 469, Berndt Hall, Public Health Department, Health Sciences Division, Fort Lewis College, Durango, CO 81301 USA; 2 Southwest Institute for Research on Women, College of Social & Behavioral Sciences, Family & Community Medicine, College of Medicine, University of Arizona, Tucson, USA; 3 Department of Applied Health Science, Prevention Insights, Indiana University School of Public Health-Bloomington, 809 E 9th Street, Room 101, Bloomington, IN 47405 USA; 4 Department of Applied Health Science, Indiana University School of Public Health-Bloomington, 809 E 9th Street, Room 203, Bloomington, IN 47405 USA; 5 Department of Applied Health Science, Indiana University School of Public Health-Bloomington, 801 E 7th Street, Room 102, Bloomington, IN 47405 USA; 6 School of Public and Environmental Affairs, Indiana University, Room 351, 1315 E 10th Street, Bloomington, IN 47405 USA

## Abstract

**Background:**

Globally, and in India, research has highlighted the importance of community engagement in achieving national vaccination goals and in promoting health equity. However, community engagement is not well-defined and remains an underutilized approach. There is also paucity of literature on community engagement’s effectiveness in achieving vaccination outcomes. To address that gap, this study interviewed Indian vaccination decision makers to derive a shared understanding of the evolving conceptualization of community engagement, and how it has been fostered during India’s Decade of Vaccines (2010-2020).

**Methods:**

Semi-structured interviews were conducted with 25 purposefully sampled national-level vaccine decision makers in India, including policymakers, immunization program heads, and vaccine technical committee leads. Participants were identified by their ‘elite’ status among decisionmakers in the Indian vaccination space. Schutz’ Social Phenomenological Theory guided development of an *a priori* framework derived from the Social Ecological Model. The framework helped organize participants’ conceptualizations of communities, community engagement, and related themes. Inter-rater reliability was computed for a subsample of coded interviews, and findings were validated in a one-day member check-in meeting with study participants and teams.

**Results:**

The interviews successfully elucidated participants’ understanding of key terminology (“community”) and approaches to community engagement propagated by the vaccine decision makers. Participants conceptualized ‘communities’ as vaccine-eligible children, their parents, frontline healthcare workers, and vaccination influencers. Engagement with those communities was understood to mean vaccine outreach, capacity-building of healthcare workers, and information dissemination. However, participants indicated that there were neither explicit policy guidelines defining community engagement nor pertinent evaluation metrics, despite awareness that community engagement is complex and under-researched. Examples of different approaches to community engagement ranged from vaccine imposition to empowered community vaccination decision-making. Finally, participants proposed an operational definition of community engagement and discussed concerns related to implementing it.

**Conclusions:**

Although decision makers had different perceptions about what constitutes a community, and how community engagement should optimally function, the combined group articulated its importance to ensure vaccination equity and reiterated the need for concerted political will to build trust with communities. At the same time, work remains to be done both in terms of research on community engagement as well as development of appropriate implementation and outcome metrics.

## Background

The Global Vaccine Action Plan 2011–2020 lists equity as one of its six guiding principles [1]. Resonating this ethos, various national vaccination policies and programs have acknowledged vaccines’ contribution to preventing high-cost treatments and averting medical impoverishment, while striving to extend the benefits of immunization to all [2, 3]. Correspondingly, community engagement (CE) for vaccinations has increasingly been recognized by decision makers [4] as a core component of working toward health equity, with a focus on community-based participatory research [5–7]. CE has been lauded for its facilitation of research translation [8] and for fostering positive perceptions of vaccines and immunization-related interventions [9], while decreasing the likelihood of therapeutic misconception [10]. CE also has been recognized for its assertion that research and interventions with people but without their input is unethical [11]. Further, recurring incidents of vaccine backlash by communities, as demonstrated by skepticism, resistance, and lack of vaccine support, are often attributed to ‘inappropriate CE’ [12]. Despite this salutogenic understanding of CE, which has been hypothesized to be a pathway through which population health goals related to public health equity can be met [13], several studies have suggested that CE has not been clearly defined or explicated in the context of vaccination programs [14, 15]. It is important to understand how CE may be utilized to ensure that vaccines are translated into affordable and globally accessible public health solutions, which are acceptable by all communities [16–18].

To do so, this study examined CE in the context of India’s Universal Immunization Program. India has made tremendous progress during the “Decade of Vaccines” (2010-2020) by introducing multiple new vaccines along with striving to increase access to new and underused vaccines in the country [19]. The National Vaccine Policy of India mentions ethical use and equitable access as its basic mantra (Ch 5, p 28). However, vaccine decisionmakers are increasingly concerned with the 62% vaccination uptake prevalence among vaccine-eligible children (12-23 months), compared to the 90% target set under government’s Universal Immunization Program, to be achieved by the end of 2020 [20].

Vaccine studies also indicate the need to embed CE within India’s immunization programs [19, 21, 22]. This growing sensitization about CE among Indian vaccine decision makers has been bolstered by the Supreme Court advisory which recommends meaningful dialogue with communities to accelerate vaccination uptake [21]. CE is also perceived to be an important step in addressing communities’ vaccine resistance, which leads to delays that inhibit timely vaccination. For example, the cervical cancer-preventing human papilloma virus vaccine was suspended by the Supreme Court of India in 2010. Later, the country's right-wing groups wrote to the Prime Minister expressing concerns about pharmaco-governance and asserting that foreign companies were pushing the vaccine onto an unsuspecting public. Through July 2020, despite advice by the National Technical Advisory Group on Immunization, the Federation of Obstetric & Gynecological Societies of India, and the Indian Academy of Pediatrics for its inclusion in the Universal Immunization Program, substantial community resistance remains. As a result, the vaccine has been sporadically rolled out in three (Sikkim, Punjab and Delhi) out of 36 states and Union Territories [23, 24].

Community skepticism about vaccines has a long history in India, evidenced by covert and overt vaccine resistance. As early as the mid 1800’s, some Hindus resisted the smallpox vaccine on religious grounds, because the material used for vaccines was drawn from the lymph of a cow, which is considered a sacred animal by the community [25]. During the National Polio Surveillance Program, community resistance ranged from people closing their doors and windows when they heard vaccinators approaching their houses, to vaccine backlash such as physical conflict between vaccinators and communities [26]. Recently, in 2017, there was decreased uptake of measles-rubella vaccination in certain Indian states amidst community uproar following social media rumors of political conspiracy and safety concerns about the vaccine [27, 28]. Thus, work to articulate a shared conceptualization of CE is critical at this juncture to establish concerted and strategic CE that can facilitate transparent vaccine communication between communities and decisionmakers and build on existing technology, interventions, and healthcare systems to address inequities in vaccination coverage, especially among under-reached and underserved populations. This may be especially useful in overcoming communities’ myths and fears about new vaccines, which are often considerably more expensive than existing ones, and target relatively ‘hidden’ diseases [21, 23]. However, current CE evidence is limited to a few systematic examinations focused on community counselling and vaccination campaigns, often in pockets of high vaccine resistance and low vaccination coverage [29, 30]. These reviews have also focused on public opposition rather than involvement, and no data have been collected to indicate ‘if’ and ‘how’ communities are engaged beyond individuals’ decisions to vaccinate themselves and their children [31–33]. The wider body of academic literature attributes this dearth of CE related studies to the variously premised and sometimes conflicting definitions of and rationales for CE [34] and the absence of CE metrics [35]. Other studies mention that evaluating CE is challenging, as such activities often occur in the context of ongoing work and throughout the process of adopting more collaborative engagement approaches [36, 37].

It is our perception that typifying an understanding of CE may lead to contextual and ethical application of CE within a complex system of relationships among researchers, policymakers, implementation scientists, and vaccine users. It may also prevent erroneous assumptions about its value and utility, or lack thereof, and inform research and data needs related to CE. This may, in turn, trigger a policy dialogue focused on robust measures to assess what works, how it works, and, over time, if CE efforts have improved vaccination rates and thereby bolstered national efforts to reach out to every vaccine eligible child and adult. Therefore, this study aimed to examine elite Indian vaccine decision makers’ individual perspectives and collective understanding about CE, the circumstances in which CE has been implemented, and how they have fostered CE for effective vaccination.

## Methods

Schutz’s Social Phenomenology Theory was used as an underlying approach because it is consistent with the belief that ‘conceptualizations’ are socially constructed and appropriated to explore participatory action [38]. This theory also helped direct attention toward considering the dynamic contexts in which CE was conceived and operationalized [39]. Social Phenomenology further helped to treat CE conceptualization and its fostering as intersubjective, integral to institutions and systems, all embedded in history, time, and space [40]. The lead author’s (TD) professional role was that of a translational researcher, supporting evidence-based programs and policy through examination of ecological frameworks using a community-based participatory approach. Her *a priori* assumption was that community engagement can foster social, relational, and ethical progress toward health equity [41]. However, few assumptions were made about how decisionmakers would conceptualize CE and community, as these issues infrequently are described in formal, written documents and must instead be intuited from distally related activities.

In preparing for this study, the lead author (TD) purposefully identified 30 individuals who had authoritative roles related to vaccine discovery, development and delivery, such as national-level vaccine decision makers who were policymakers, program heads and/or associates in the government, private sector, non-governmental organizations, and country-offices of international donor and UN agencies. Thus, these individuals, by virtue of their knowledge and positions, were the ‘elites’ [42] and were able to provide a unique ‘big-picture perspective’ [43] about CE strategizing and implementation during India’s Decade of Vaccines. Interviewees were approached with this status differential in mind [44]. In keeping with the assumptions and beliefs of social phenomenology, a two-step participatory approach for data collection was used: (1) semi-structured elite interviews followed by (2) a member check-in meeting [42]. All interactions used a community-engaged approach, including emphasis on mutual respect and recognition of the knowledge and expertise of study participants. This included adhering to participants’ preferred meeting dates on December 25 and January 1, even though these were national holidays. Further, the member check-in meeting was democratically conducted rather than using the researcher as a moderator. In addition, the researcher was sensitive that issues related to vaccine resistance were occurring in real time, wherein trust building with the study participants was necessary to obtain ‘good data’ and completion of the project.

Access to participants (distinct from identification of the sample) was obtained using a snowball methodology, beginning from the professional network of the principal investigator (TD). Recruitment emails were sent by TD in December 2017 to each of the 30 potential participants, followed up with phone calls to identify interest and availability for an in-person interview. Interviews were conducted by TD with 25 individuals who agreed to participate in the study from December 2017 to February 2018. Each interview lasted for 50 to 90 minutes, was carried out in English, and conducted in the country-offices of the respective agencies, institutions, and organizations located in or around New Delhi, the capital city of India. Interviews were audio recorded and transcribed verbatim. All personalized information was anonymized. Data also included field-notes written within 24 hours of each interview. The interview topics drew from earlier studies focusing on CE as a strategic tool for vaccine research and rollout [45, 46]. Accordingly, the inquiries explored participants’: (i) conceptualization of community and CE, (ii) evolution of CE, (iii) fostering support for CE, (iv) resources available for CE, (v) partnerships for CE, (vi) enablers to CE, and (vii) barriers to actualize CE. The interview guide used for this study, including questions and probes, is available as a digital supplement (1) to this article.

Once a preliminary analysis of the interview data was completed, TD presented the findings in a one-day member check-in meeting among the study participants and their teams (who held second-line leadership positions) in January 2018. Study participants and their team members who participated in the member check-in meeting were knowledgeable about the issue and were comfortable validating and candidly critiquing the primary findings. All study participants and their teams were nationally known; thus, in order to maintain confidentiality, identities, names, and organizational affiliations were not used in reporting the findings. Therefore, although participants in the follow-up meeting knew each other, no specific responses were linked to any individual or organization. This meeting ensured that the overall summation and meaning making of the findings prepared by TD and the research team conformed with what the study participants had mentioned in their interviews and made sense to both the vaccine decision makers and their teams in India (e.g., validity). This was a participatory way to verify both data saturation and completeness of the findings, as well as archival document review (part of the overall project, but not of this study). The study was approved by Indiana University’s Institutional Review Board.

### Data analysis

First, all data were transcribed verbatim and entered in NVivo12 (QSR International, Melbourne, Australia) for qualitative data management.

An *a priori* coding structure was used to categorize individual participants’ conceptualization of CE, how their interests in CE for vaccination evolved by overcoming barriers and optimizing facilitators, while integrating 'policy push for vaccine uptake' and 'generating vaccination demand pull' approaches for different vaccines under the UIP. Based on the interpretive analysis used in social phenomenology, first-level broad construction of CE was done, followed by second-level typical constructs, deliberated through critical events or performance of CE ‘duties’ and ‘responsibilities’ throughout the tenure of the decision makers [47]. Categories conceptually corresponded with the Social Ecological Model, which has been used to study vaccination uptake and health disparities [41]. Given this loose pre-existing framework, a general inductive approach was used [46]. To reach intercoder-reliability (>90%), two coders joined TD, iteratively reviewed, and re-reviewed data for existing and emerging themes and/or patterns, and ultimately crystallized a holistic interpretation through multiple coding conferences. Thereafter the three coders independently coded five interviews to test, reject, accept, or refine the codes [43]. The final coding structure contained 7 multi-dimensional CE themes with 42 nodes. Exemplar interview excerpts illustrate the findings, although the analysis drew from the entire dataset. The coding structure is available in full as a digital supplemental (2) file.

## Results

All study participants held national and multi-regional leadership roles in vaccine policymaking, financing, and/or program planning and management across vaccine research, development, and roll-out stages for at least ten years in India. In addition to their roles in India, five participants reported managing programs in multiple countries in Asia, Africa, and Latin America. Table 1 describes the study participants.

**Table 1:** Profile of vaccine decision makers in India who participated in this study, 2018

Study participant	Academic background *(Basic science and research = Basic Science; Public Health/Community Medicine/, Humanities/Management = Humanities/ Public Health)*	Category of organization of employment *(Govt. of India=GOI, Technical body of the GOI = Technical body, UN organization, Donor organization = Donor, National level NPOs with policy influencing and vaccine roll out role = NPOs (Policy influencing), International /National Level NPOs with vaccine research and roll out role = NPOs (Research, roll-out, advocacy)*	Governance levels *MoHFW= Ministry, State level nodal institutions of the Ministry = State nodal office, Technical consortium under the aegis of the Ministry= Technical consortium, HQ in a developed country with country office in India = Country office in India, Principal financial recipient from a foreign donor and programmatic ownership of Govt. of India = Principal recipient projects, NPOs with an India office)*	Organizational role for community engagement *(Establish regulations = Regulatory, Carry out surveillance/research= Surveillance/research, Provide funding = Financial support, Develop policy guidelines/technical support = Technical support, Develop communication strategies and materials= Communication strategies, Implement nationally sanctioned policies and programs = Policy and program implementation)*	Leadership’s level of decision making *(Asia-Pacific region, National level, State level)*
1.	Basic Science	GOI	Ministry	Regulatory	National level
2.	Basic Science	GOI	Ministry	Regulatory	National level
3.	Basic Science	GOI	State nodal organization	Policy and program implementation	State level
4.	Humanities& Public Health	GOI	State nodal organization	Policy and program implementation	National level
5.	Basic Science	GOI	State nodal organization	Policy and program implementation	National level (Retired)
6.	Basic Science	Technical body, GOI	Technical consortium	Technical support	National level
7.	Basic Science	Technical body, GOI	Ministry	Policy and program implementation	National level
8.	Basic Science	GOI	Ministry	Regulatory	National level
9.	Basic Science	GOI	Ministry	Regulatory	National level
10.	Basic Science	UN organization	Country office in India	Regulatory and Surveillance/research	Asia-Pacific region
11.	Basic Science	UN organization	Country office in India	Technical support and Communication strategies	Asia-Pacific region
12.	Basic Science	UN organization	Country office in India	Regulatory	Asia-Pacific region
13.	Basic Science	Donor	Country office in India	Financial support & Technical support	National level
14.	Basic Science	Donor	Country office in India	Financial support & Technical support	National level
15.	Basic Science	Donor	Country office in India	Financial support	National level
16.	Basic Science	NPOs (Policy influencing)	Principal recipient projects	Communication strategies & Policy and program implementation	National level
17.	Basic Science	NPOs (Policy influencing)	Principal recipient projects	Communication strategies & Policy and program implementation	National level
18.	Basic Science	NPOs (Policy influencing)	Principal recipient projects	Communication strategies & Policy and program implementation	State level
19.	Humanities/ Public Health	NPOs (Policy influencing)	Principal recipient projects	Communication strategies & Policy and program implementation	National level
20.	Humanities/ Public Health	NPOs (Research, roll-out, advocacy)	NPOs with India office only	Surveillance/research & Technical support	Asia-Pacific region
21.	Basic Science	NPOs (Research, roll-out, advocacy)	NPOs with India office only	Surveillance/research & Technical support	Asia-Pacific region
22.	Basic Science	NPOs (Research, roll-out, advocacy)	Country office in India	Regulatory & Surveillance/research	Asia-Pacific region
23.	Basic Science	NPOs (Research, roll-out, advocacy)	Country office in India	Surveillance/research & Technical support	National level
24.	Humanities/ Public Health	NPOs (Research, roll-out, advocacy)	Country office in India	Policy and program implementation	National level
25.	Science	NPOs (Research, roll-out, advocacy)	Country office in India	Policy and program implementation	National level

This section sequentially shares results organized by the following categories and subcategories. Because the results are extensive, we list many of the key themes in brief here as well.

1. conceptualization of community, and how stakeholders define community;
  - community was typically understood to be one or more of the following: vaccine-eligible children and their parents and vaccine-eligible adults, frontline healthcare providers, local-level stakeholders, vaccine gatekeepers, and local-level implementing organizations.
2. conceptualization of CE, with particular attention to analyzing extant efforts, which generally fell into three categories:
  - capacity building of frontline stakeholders as CE;
    i. capacity building most often was expressed as training, training-of-trainers, and course offerings;
  - vaccine-related information dissemination as CE;
    i. participants described a wide variety of different communication methods, as well as perceived benefits and disadvantages to each;
  - targeted community interventions as CE;
    i. participants provided examples of ways in which community
      interventions had been carried out;
3. different tangible ways in which CE might be fostered;
  - fostering CE was viewed on a broad spectrum that ranged from highly participatory approaches to direct imposition of vaccination services;
4. evolution and transformation of CE;
  - all participants acknowledged the need for a better understanding of CE and, in the member check-in meeting, came to a consensus on a definition of CE.

### Conceptualization of community

Most participants defined communities as ‘beneficiaries of the UIP,’ with a notion of transactional exchange of vaccine related information between the providers and the communities, always with the aim of vaccination uptake. Communities consisted of the following categories of people: (1) vaccine-eligible children, vaccine-eligible young adults, and their parents and guardians who make vaccination-decisions for the former; (2) healthcare providers who deliver vaccines and sensitize vaccine-eligible populations and their guardians for improved vaccination rates and herd immunity; (3) local-level stakeholders who disseminate information to encourage vaccination uptake; (4) gatekeepers who resist a particular vaccine or vaccination *per se*, and; (5) local-level implementing organizations of community health workers, groups that includes what are known in India as the 3As. These are Auxiliary Nurse Midwifes who are based at a sub-center and are multipurpose workers responsible for administering vaccines among communities of < 5000 people; Accredited Social Health Activists, who are local women trained to act as health educators in their communities, catering to 700 people in tribal areas and 1000 in rural villages; and Anganwadi Workers, resident workers in the village rural child care centers in India who are responsible for promoting maternal and child health, including interpersonal communication for full immunization coverage, among communities of <1000 people. A few participants took a broader perspective: “*It is the whole communities in which those individuals were living.”*

Most of the participants acknowledged their distance from the community, mentioning *“if I went to the community nobody will accept me,”* while comparing the sense of community with local organizations because they *“help raise community demand for routine immunization.*” These organizations included grassroots non-profit organizations (NPOs), community-based organizations (CBOs) like women’s self-help groups, local-level representatives of occupational groups like brick-kiln workers and barbers, and the local-chapters of technical and youth organizations such as the Indian Association of Pediatricians and Nehru Yuva Kendras Sangathan (autonomous organization for youth development under the Government of India, Ministry of Youth Affairs and Sports). Several NGO heads identified themselves as ‘communities’ for their people-centric approach, though, in most of these expressions, fractious relationships and issues of incompatibility between decision makers [mostly government or donors] and NPOs were evident.

*“ … .they* [Government or donors] *want to clip our wings. This is very sad because we* [NPOs] *bring up issues* [local issues of the communities]*, which you* [Government or donors because of being at the national-level] *might never know.”*

Some participants identified vaccine-gatekeepers, people who were suspicious that vaccination is a political agenda against minority groups, as communities. Interventions targeting their positive vaccination decisions came across as an area of CE.

*“ … in Mallapuram the mother generally said ‘no’ to vaccination because their husband lived in the Middle East* [who was proxy decision-makers for their child’s vaccination]*.”*

Finally, it was unclear whether the media was part of the community, or a driver of communities’ vaccination decisions. Most participants indicated that the media spread misinformation and promulgated negative sentiments among vaccine priority populations about vaccines, and thus expressed the need *“to stop negative media so that they* [media] *do not “blindly publish”*, or “*over-sensationalize when it is not an Adverse Event Following Immunization.”*

### Conceptualization of CE

The participants perceived CE both as a strategy and tool in implementation terms, and variously defined CE as segments of processes comprising of: (1) vaccine policy and program formulation; (2) capacity-building of frontline stakeholders; (3) vaccine information dissemination among communities to promote vaccination uptake, and; (4) targeted community-level interventions to curtail the recurring incidents of vaccine-related community backlash. There was evidence of relational goals of CE, like *“longer-term trust building”* [between the vaccine decision makers and the communities], and *“ … .understand what is going on in people’s minds* [regarding vaccinations]*”*.

Intuitively, all the participants proposed ongoing and early instantiation of CE for better vaccination outcomes:

*“We always go to the communities earlier and have media campaigns, and interpersonal communications to sensitize people on what* [vaccine] *we would give to their children.”*

However, several participants critiqued that CE interventions came in waves, mostly during vaccine introductions, before and during vaccine trials, and in response to a disease outbreak. They also noted that there were no tools or metrics to measure its impact. They speculated that these deficits may be because:

*“The Immunization Technical Unit was not built with a CE model* [CE frame] *for immunization. Like, you* [Government] *compensate Accredited Social Health Activists for fully immunizing children and trainings attended, but not for doing CE.”*

Participants described a top-down and decentralized vaccine governance structure where vaccine policy formulation and vaccine introductions were conducted at the Ministry, considering disease burden, vaccine cost, cold-chain, and supply chain issues. These efforts were completely funded by the Ministry of Health and Family Welfare (MoHFW) and international donors.

*“ … .*[CE is like] *a chandelier, the [MoHFW] is the hook. The different lights are the different partners, they are held at right distances in the right manner. In immunization, the roles and partnerships* [of national level decisionmakers] are clearly defined*.”*

The development of vaccine policies and operational guidelines in English and Hindi (one of the 22 scheduled languages of the Republic of India, and also one of the official languages of India which is understood, spoken, and read by more people than English) by the technical bodies of MoHFW, such as the Immunization Technical Support Unit, and the Mission Steering Group, was conceptualized as CE too. Participants mentioned that the “*state translated and modified* [these documents] *if they think that something is to be added or deleted,”* though no such example of any such revisions incorporated based on communities’ recommendations was cited.

Except the *Vaccine Policy (2011),* which recommended enhancing communities’ vaccination acceptance and confidence, and vaccine-specific *Operational Guidelines,* which recommended community-facing strategies, participants did not identify any sub-population-based CE-specific policy. Almost half of the participants cited the *Communication Strategy for Polio Eradication*, published by the UNICEF and USAID CORE Group, detailing intensive outreach for polio vaccination, as nearest to any CE guideline. Three participants, considering India’s diversity where *“every mile the language changes, the culture changes”* suggested having a *“village-level communication strategy.”* Participants noted strategic programs like Mission Indradhanush and Intensified Mission Indradhanush to achieve 90% immunization *“to the last child”* as CE.

The heads of organizations and technical bodies often criticized chasms in this one-way, top-down approach as “*working in silos”* and *“not real CE,”* and feared that it would ultimately *“hinder an integrated approach.”* A few participants identified CE as activities occurring in spaces like Village Nutrition and Sanitation Days, which are organized monthly at rural childcare centers. There, communities can ask questions about vaccines and vaccination strategies. However, these participants were doubtful that communities possessed any emancipated voice beyond seeking or resisting vaccines.

### Capacity building of frontline stakeholders

Some participants mentioned ‘cascade training of trainers’ for the 3As and local Master Trainers as CE, since the goal is to motivate communities for full immunization. Notably, the CE roles of the 3As and other local stakeholders were different. The Auxiliary Nurse Midwives and Anganwadi Workers are salaried staff for vaccine administration among communities and the Accredited Social Health Activists receive honoraria for counselling and escorting the communities to vaccinations. However, the local NPOs and CBOs appeared to be instrumental in carrying out community-based activities to motivate each community’s vaccination decisions, and, in the case of vaccine trial conducting organizations, act as conduits between researchers and vaccine clinical trial participants.

Participants conceptualized the 18 months training for ANMs, and 3–4 weeks trainings for AWWs and ASHA workers respectively, with additional trainings such as the 3-day Boosting Routine Immunization Demand Generation course for the 3As, and vaccination sensitization trainings for the local-level vaccine-champions (community advisory boards, local religious leaders, barbers, and CBO members), as CE. In these instances, it appeared that some interpersonal tactics were imparted to frontline stakeholders, and tasks were later delegated to them. However, a few participants questioned the ‘quality of CE outcomes’ from these trainings:

*“So, you* [Government] *piggy back everything on the Community Healthcare Worker, who talks to communities about everything: immunization, family planning, maternal health, school health, adolescent health, non-communicable diseases, and cancer* … [but] *you are not actually engaging or doing CE.”*

### Vaccine-related information dissemination

Most respondents mentioned “*bilateral information transfer* [interpersonal and behavior change communication] *sent down to communities”* as CE. In the same vein, most participants denoted the Communications Officer as the CE human resource. In fact, one participant said, *“The role of communication, I mean CE, sorry using the wrong word again.”*

Some participants highlighted the need to be creative and explore web-based media, considering its ease of use, cost-effectiveness, and penetration to interior locations:

*“Nobody is interested to read your mobile texts. So, use GIF messaging.”*

There were a few examples where bottom-up information, going from the community to the government which facilitated realizing the vaccine program goals, was acknowledged:

*“In a construction site we* [participant’s organization] *did the mapping. But when we reached the community after a fortnight, they* [community] *have already migrated. The local person would tell us the whereabouts of the mobile community and we could then reach them through the Accredited Social Health Activist network.”*

Some participants highlighted campaign-related booklets like the area-based ‘*Underserved Strategy,’* developed after a polio outbreak in Uttar Pradesh in 2002 among the Muslim populations, the ‘*Social Mobilization Network’* formed in 2001 to sensitize families to polio immunization, ‘*My Village my Home’*, a pictographic vaccination tracking method in the shape of a hut, where each column of the hut contains vaccination details of each new-born in the village, and media trainings of *“State Immunization Officers on how to handle the media and stop negative media,”* as CE.

Vaccine champion engagement and celebrity engagement to motivate communities’ vaccination decisions came across as another form of CE, though there were mixed reactions regarding this strategy.

*“Our communication campaigns are pathetic. What is the point in having* [a film star in his 70s*] there? We have no way of measuring CE. Does he convey safety of the product? To sell a toothpaste or a phone we spend hundreds of millions of dollars. How much is going into selling something far more important as vaccines?”*

### Targeted community interventions

Some participants perceived CE as a [right of the communities], *“communities want the leadership to come to them. … just sit with them* [communities], *work with them and that is CE. The leader needs to go to the community at least once or twice. It really increases the communities’ motivation and trust.”*

Others suggested a more emancipatory understanding of CE:

“[Vaccine] *demand generation is another thing* [than CE]*. It means that you* [government/vaccine providers] *are giving and we* [vaccine-eligible community] *are accepting. Policy influencing is that where the* [empowered] *community thinks that certain things needs to be changed* [and advocates for that]*.”*

Intervention programs reflected a range, between vaccine imposition and respectful engagement with community stakeholders, where participants’ responses reflected balanced trade-offs between CE’s time and resource investments and feasibility, emphasizing that it is a “*marathon, and not a sprint,” “an expensive process” and “took 20 years to learn about community and how to do CE.”*

*“In XXXX district community was very resistant and started beating the vaccination team. Then we had to contact a local muscleman, briefed him that this* [carrying on with the vaccination drive] *is important, and then told him to make an announcement that vaccination is not a bad thing.”*

*“We engaged with the staff of Aligarh Muslim University, Jamia Milia Islamia and Jamia Hamdard* [institutions of higher education that were created to manifest indigenous ethos and spirit of diversity in India]*, who went to the field. That helped to address the issue of vaccine hesitancy among religious leaders* [especially the Muslim religious leaders]*.”*

Later, in the member check-in meeting, participants reiterated that effective CE conceptualization and conduct will require developing CE performance and outcome indicators and advocating for their incorporation in immunization surveillance instruments in India. Herein, all the participants emphasized the need to document CE effectiveness and its relational gains:

*“ … as a country, I will be ashamed … ., very poor in documentation. You will hardly see any papers from the learnings of polio eradication. This is so because the people who are doing CE do not have the time to document.”*

### Range of approaches to fostering CE

Though a strict categorization of responses by organizations would not be accurate, participants endorsed a wide variety of types of approaches to fostering CE. These methods generally fell on a spectrum ranging from empowered (‘1’) to disempowered (‘7’). Table 2 provides exemplar quotes illustrating efforts or actions that might be categorized into these different levels.

**Table 2:** Broad categorization of ownership and fostering of CE by vaccine decision makers in India, 2018

	Categorization of ownership and fostering of CE by vaccine decision-makers	Exemplars
1.	CE as a community empowering role	*“Some journalist misinterpreted and had adverse reports. Honestly, we didn’t get that much support from authorities in my headquarters but my CAB met within few days and went ahead and said that we are willing to give out rejoinder to this news report because we know you have been very very meticulous about protecting the individuals, have been transparent and sensitive to community.”*
2.	CE as vaccine delivery with the help of frontline workers	*“I always appreciate my workers. The hard work that our ANMs and field staff are putting in in is tremendous. So, if we have coverage of 90%, it is not my contribution, it is all because of my field workers who are doing a great job”*
3.	CE as a part of the organizational structure	*“There was the country and the regional programs where there were advocacy efforts to engage the community. There was a representative in the senior management team from the CRP. Representatives of the Board of Directors, who in one capacity or the other were the advocates of the community.”*
4.	CE as a proactive social and altruistic responsibility	*“I remember there were two places in Tripura where the vaccines were transported through helicopter. First time Government of India gave that fund. Although the beneficiary children were only 15 I argued that if 100% children are to be immunized you have to somehow send the vaccines to this remote place. Else, it will take 7-8 days to reach there.”*
5.	CE to comply to GOI and/or global mandates	*“Since we did not have any great experience in vaccine trial and community engagement it* (CE) *was introduced to us through our sponsors and collaborators like National Institute of Health”*
6.	CE as a duty delegated to the States and lower offices	*“No no, we do not do that part. Government of India does not run the programs at the peripheral levels.”*
7.	CE as vaccine imposition/delivery	*“When children were dying and Japanese encephalitis vaccine was introduced, people were fighting to get the vaccine. There was a firing in three places. People got a notion that if the vaccine stocks were finished, we will go and their child will not get vaccinated, and so the rush and the panic ‘me first’ ‘me first.”*

All participants acknowledged “*decision makers’ good intention for CE but they were not matched with recipes of successful CE models.”* Most of the CE interventions reported occurred during the National Polio Surveillance Program (a campaign of the World Health Organization and MoHFW initiated in 1995 to ensure polio eradication through house-to-house poliovirus vaccine delivery), with minimal evidence of institutionalization, replication, or scale-up of these during introduction of other vaccines.

### Evolution and transformation of CE

All participants indicated that CE was still a *“very poorly understood space,” “complex,”* and there were *“several gaps to understand this puzzle.”* Three participants from NPOs critiqued that it is “*offhand,” “ad-hoc practices to douse the fire,” “firefight,”* or *“control big chaos and help put things back to normal”* and recommended *“real community engagement”* and a *“scientific approach to CE.”* Recollecting CE’s evolution, participants noted that the earlier paternalistic prevention impositions has built a negative community memory, and jeopardized communities’ trust on vaccine authorities:

*“..the vaccine fear was connected to the family planning program wherein women were forcibly sterilized.”*

There was some evidence of pragmatic pressures by global provider/donor organizations (e.g., *“GAVI funding went partly for community mobilization”*) that reinforced renewed systems-thinking and inclusive bottom-up- models, like:

*“We were not really very serious and formed a small community group.* [Initially, the community group*] came, had some snacks and went off. CE really didn’t go beyond that. But by then the NIH and USAID wanted Community Advisory Boards or CABs … and then we learnt how necessary it was.”*

Consequently, several participants described recent and direct interactions between vaccine decisionmakers and communities while referring to “*The Prime Minister’s Office invites suggestion from the public”* and “*Health Minister issues letters to each Accredited Social Health Activist and Auxiliary Nurse Midwife encouraging them to vaccinate every child.”*

In the day-long member check-in meeting, the summary of analysis from the interviews was presented. Study participants and their teams agreed with the findings, and jointly came up with a robust definition of CE, which can be summarized as:

*“CE is an upstream policy imperative rather than downstream interventions to build trustworthy relationships between vaccine decisionmakers and communities. It involves demystifying vaccine science and transparent communication for empowered community agency. This would enable communities to critically analyze vaccine related myths and misinformation and enable knowledge co-production in building community sensitive vaccine policies and programs.* [CE] *is incumbent to sustained political-will and resources to ensure evidence-informed, tailored, vaccine policies and programs, providing equitable, quality, and tangible vaccination and capacity building benefits to community members.”*

Meeting participants recognized the need to carry out interventions in ways such that trustworthy relationships between communities and decision makers are established. There were comments reflecting realizations like *“If we* [decisionmakers] *close the doors once again to the community, we might lose their trust, and not get the communities back, ever again.”* They also recommended creating more opportunities for relationship-building and group discussions between community healthcare workers and vaccine decision makers. Meeting participants were especially interested in addressing inequities in vaccination coverage by building on the existing range of interventions while innovating newer mechanisms such as community mobilization for vaccination, strategic interventions with vaccine gatekeepers, providing immunization information using traditional, digital, and social media, and dispelling vaccine misinformation and disinformation while formulating rumor management strategies.

## Discussion

This study was able to identify elite decision makers’ core conceptualizations of community, CE, and both extant and aspirational approaches to CE related to vaccination programs in India. In reviewing these findings with study participants, a core definition of CE emerged, focused on upstream relationships (bidirectional), fostering trust, transparent communication, capacity building, and political will to ensure such approaches. Participants indicated that much of the extant work being conceptualized as CE is primarily downstream delivery and even imposition of services for vaccination uptake. While such things can be beneficial (e.g., vaccination), it likely matters to whom they done, in what way, and with what level of community voice (e.g., changing “to whom” to “with whom”). Given that direct imposition has resulted in community backlash against vaccination campaigns both in India and other parts of the world, including violence and hiding children from vaccinators, achieving national policy goals and fostering equitable distribution of public health outcomes may be difficult without a revised approach to CE. Concomitant to this must be an increased focus on CE metrics to promote greater understanding of processes and goals. Importantly, each of the different approaches to CE, including direct imposition, appeared to have been done with the primary goal of increasing equitable access to vaccinations (e.g., supporting community immunization). Thus, the underlying question discussed in this study did not focus on *whether* individuals should have equitable access to vaccinations, but rather on *how* such an outcome might best be achieved – that is, the degree to which a revised understanding of CE can support bilateral improvements in both vaccination dissemination by the government and vaccine confidence among communities.

Notably, being an Indian but performing the research at an American university, mitigated reflexivity issues and gave TD the identity of an 'informed outsider,' which allowed her to gain increased access to elites [43]. Being an implementation researcher allowed TD to deeply engage in analyzing the data while utilizing NVivo predominately as a data management tool. The member check-in meeting facilitated a participatory approach to the interviews, providing considerable interpretive latitude, and probing opportunities. It also allowed participants to critically review CE in UIP with a diversity-equity-inclusion focus. This was particularly important because studies on ‘elite interviewing’ mention that such access can be rare, because such people are hard to reach, surrounded by gatekeepers, and have power and ability to protect themselves from intrusion and criticism [44, 48].

This study also benefited from the fact that none of the CE strategies/interventions were ranked as ‘best practice’ over another by institutional mandate or leadership, unlike the traditional ranking of engagement models in Holland Matrix (1997) [49], or Arnestein’s Ladder [50]. This helped reduce social desirability issues among the participants, who would not be perceived as ignoring a best-practice approach when answering honestly about CE.

In most cases, decision makers did not identify themselves or their families as ‘community’, and in some cases only a section of the public was perceived as ‘community’. Ensuring full immunization to communities under UIP was considered the most important CE goal and a step toward equitable health outcomes. However, as noted in other literature [35, 51], a non-immersive and reductionist approach to conceptualizing communities may inhibit formation of trusted collaborations with the communities, ultimately compromising the creation of communities’ agency [52]. Some authors have described this as ‘conservative corporatism’ which, contrary to the ‘whole community approach’ [53], can lead to fragmented health governance, introduce barriers to building comprehensive people-centered vaccine policy reform [36], and risk defining communities as internally homogenous entities, which is unlikely to be the case given the diversities prevalent in India [54]. This may also undermine tailored CE strategies for particular sub-populations, leading to reductions in their trust of vaccinators and empowered vaccination decision making, especially among those for whom vaccine hesitancies are high, and/or vaccination uptake is low [55, 56].

While findings supported current iterations of CE in making substantive contributions to vaccine demand generation and disease eradication, communities were often seen as offering ‘passive demand.’ Ideally, communities would actively seek vaccines and there would be community demand reflecting social support for vaccination as a norm [11]. Head’s research goes so far as to suggest that utilitarian CE may foster health inequities [57]. Gopichandran’s work looks at the relational gains (more intrinsic in nature, rather than transactional relationship building) from CE and posits development of trust between vaccine decision makers and communities as a result of shared CE goals integrated into vaccination targets [56]. Accordingly, doing empowered CE may require a paradigm-shift to perceive communities as integral parts of the policy and delivery systems, incorporate CE metrics into vaccine surveillance, and create new roles with a focused responsibility to coordinate CE.

It appears that many facets of the national-level CE response were an equilibrating reaction to appease community outrage rather than an integral approach set in place *a priori*. Adhikari et. al. has defined such CE as 'short-hand' [31], often resulting in wasted resources, with the potential to create mistrust rather than enhance benefits, create legitimacy, or share responsibility [31, 56]. Other authors have envisaged that such CE can eventually give rise to communities as agents of the government, and CE becoming an ‘involvement industry’ ‘procured from external organizations’ [57, 58]. To alleviate this, Folayan et. al. (2019) have recommended memoranda signed between the government and local partner organizations at the study design stage [54]. That noted, Webber seems to doubt whether national government-based public health initiatives might ever be able to stray too far from a top-down approach, postulated as the ‘two-community thesis’ [58]. Other authors suggest that deviation from this paradigm will require transformative leadership which is difficult to achieve in the public service sector with the prevailing traditional organizational thinking, policies, and management techniques [59].

While frontline local stakeholders played a role in Indian vaccination efforts as two-way conduits between decision makers and the community, more studies are recommended to examine complex issues derived thereof, such as internal chasms and accountability mechanisms between the 3As, and motivational erosion when CE work is not compensated financially (adequately). Prior research would not suggest, though, that social media could replace this in-person work. Ramsbottom’s et al.’s study found that, although social-media messaging is a cost-effective mechanism for vaccine information dissemination, it might not be the best approach for India, and could leave out social media illiterate populations, those with erratic and sporadic internet connectivity, and areas where vaccine communication needs to be translated to local dialects [53].

### Limitations

Ensuring open discussion with vaccine decision makers and their team members on a potentially controversial topic like CE for vaccination was not always easy; it took time to convince the potential participants to participate. Some of the elites were difficult to access because of the ongoing community uproars around Measles-Rubella and HPV vaccines which were playing out in real time in the country during the study’s time period [60, 61]. Despite these structural impediments, theoretical saturation was ensured by virtue of interviewing nearly the entire group of elite vaccine decision makers in the country. This was achieved by utilizing TD’s professional networking and familiarity with some of the study participants, use of a sensitive mix of knowledge and intercultural humility, flexibility to re-schedule appointments after office hours or on national holidays, and use appropriately persuasive multiple communication channels like Facebook Messenger, or WhatsApp [43], in addition to emails and phone calls. Nonetheless, the study findings were limited by the inherent limitations of a qualitative study design. However, generalizability within India might be more strongly inferred than would be typical given the high percentage of decision makers who provided data. In addition, all the study participants were interviewed in or around New Delhi. While the individuals who were interviewed each had a national or international scope to their decision making, this centrality may have influenced the findings in some way. Finally, some of the findings related to intended actions in the future rather than things that had already been completed; this hampered the ability to ascribe definite actions in some cases. However, existing literature demonstrates that intentions are moderately good predictors of future behavior [62, 63].

## Conclusion

The results from this study can be used both to understand past CE challenges and successes and to prospectively plan community-led, tailored CE initiatives for better vaccination outcomes. Of note, there appears to be conceptual tension between multiple vaccination-related goals, such that each can be perceived as CE for health equity; namely, top-down vaccination programs may be successful in achieving some short-term immunization, but there may be backlash, and longer-term increases in immunization rates may suffer as a result. At this stage, it will be critical to devise CE process and outcome indicators for vaccination programs in India, and to advocate for their incorporation in vaccination surveillance datasets. As of now, the suggestions herein are theoretical – and evaluation metrics would allow for demonstrations of how CE impacts a variety of important outcomes, and, ultimately, foster replicability of successful efforts within India and internationally.

## Supplementary information

**Additional file 1.**

**Additional file 2.**

## Data Availability

All twenty-five qualitative interviews (audio recording and transcripts) are available from the lead author (TD) and can be shared on request.
