# Supplementary material for: A qualitative analysis of vaccine decision makers’ conceptualization and fostering of ‘community engagement’ in India
Source: Int J Equity Health. 2020 Oct 20;19:185. doi: 10.1186/s12939-020-01290-5 (PMC7574459; doi:10.1186/s12939-020-01290-5)
Supplement: Supplementary file 1 — Additional file 1. [file 12939_2020_1290_MOESM1_ESM.pdf]

Supplement 1: IJEH-D-20-00306R3
A qualitative analysis of vaccine decision-makers' conceptualization and fostering of 'community engagement' in India

Key informant Interview Guide

Introduction
I am Tapati Dutta, a doctoral candidate at the school of public health at Indiana University-Bloomington, U.S.A. Thank you for taking time to participate in this study. As you already know, this is for my doctoral research. I hope you received the information sheet about the study. Have you had a chance to review this?
If yes, do you have any questions about this study? [Pause to discuss questions]
If no, please take a moment to review this information (give information sheet. After review): Do you have any questions about this study? [Pause to discuss questions]

Now that you have reviewed and discussed the study information, will you agree to participate in this interview?
If no, we conclude the interview with thanks.
If yes, May I begin and start the recording?

[Start the recording]

Part I: Understanding community engagement
1. Briefly describe your institution's history with community engagement to improve vaccination uptake in India since 2010.

a. [Probe]: With which/what communities or groups does your agency tend to work?
b. [Probe]: Please describe this relationship with the abovementioned community group/s.
c. [Probe]: How was this relationship formed
d. [Probe]: How has your agency/institution engaged communities in vaccine decisions?

2. In your opinion, how does your agency/institution define community engagement?

a. [Probe]: How are the values of outreach and engagement reflected in your institutional mission statement and/or strategic plan, institution's structure and governance?
b. [Probe]: How is this similar or different from your own views?
c. [Probe]: How has this changed over time?
d. [Probe]: Is this the same across vaccines?

3. How would you think the leader or senior management in your agency/institution supported advancing community engagement for vaccines?

a. [Probe]: Can you give some examples that characterize leadership strategies and activities to advance outreach and engagement at national, state and local levels?
b. [Probe]: Would you also like to mention if there are any example since 2010 where community engagement has not been supported [appropriately] and how the leadership in your agency/institution, addressed it?

4. In what ways does your agency/institution partner with private sector, NGOs, communities and media to support and advance community engagement for vaccination acceptance and uptake?

a. [Probe]: Are these partnerships institutionally driven, or are they driven by a few leaders?
b. [Probe]: Who do you think are the leaders, advocates and gate-keepers in community outreach and engagement who are not formally charged but who are highly visible? Can you give me some examples?

Part II: Understanding the enablers and barriers of community engagement for vaccine introduction and uptake
Please describe the financial resources and trained human resources at all levels in your agency/institution to advance community engagement for vaccines?

a. [Probe]: What are the key financial and human resources for effective community engagement?
b. [Probe]: What would you think as key barriers for adequate financial and human resources for effective community engagement?

What are your thoughts on communities' level enablers to promote community engagement, especially for new and emerging vaccines?

[Probe]: Please give some examples

What are your thoughts on communities' level myths and misconceptions which are or could be barriers to community engagement for new and emerging vaccines?

[Probe]: Please give some examples

[Probe]: Please give examples of social messaging, community sensitization, community mobilization to address such misinformation and build community's trust for the vaccine delivery system and for new and emerging vaccines

In your opinion what more can your agency/institution do to build a more enabling environment within your agency/institution to advance community engagement in vaccine introduction and delivery?

[Probe]: Please give some examples

Any additional comments?
If yes [interview will continue]
If no:

We have completed the interview. Thank you for your time and the information which you provided. Once the study is completed, I will share the findings with you once we have published them.

In the course of analysis, may I contact you briefly if I have additional questions about your comments? Thanks again for your time. [Turn off the recorder].

End of interview
