# Supplementary material for: A qualitative analysis of vaccine decision makers’ conceptualization and fostering of ‘community engagement’ in India
Source: Int J Equity Health. 2020 Oct 20;19:185. doi: 10.1186/s12939-020-01290-5 (PMC7574459; doi:10.1186/s12939-020-01290-5)
Supplement: Supplementary file 2 — Additional file 2. [file 12939_2020_1290_MOESM2_ESM.pdf]

IJEH-D-20-00306R3

A qualitative analysis of vaccine decision-makers' conceptualization and fostering of 'community engagement' in India

Supplement 2: Exhaustive list of Nodes Describing Conceptualization and Support for Community Engagement for Introduction and Uptake of New and Emerging Vaccines in India, 2018. (This paper mostly utilized codes 1-5)

Conceptualization of Community Engagement	
1a. Egalitarian	Perceiving communities as equal partners and leaders by vaccine policy makers.
1b. Evaluative definition of CE	Opinion based comment on virtue of community engagement leading to vaccination uptake.
1c. Ideal normative definition examples of CE	Ideal definition of what community engagement should be, irrespective of what the organization is doing or not doing. Defining ideal community engagement processes which will lead to community engagement (goal).
1d. Organizational interventions with or for CE	The actual programmatic interventions with/at community levels by the organization for vaccination uptake.
(i) Community empowering interventions	Processes leading to empowered communities.
(ii) Personal narratives	What the respondent did in her/his current or earlier position to engage the communities. Mostly use of first person
(iii) Token interventions	Roger Hart's Ladder of Participation which defines this as the lowest level of community engagement.
(iv) Top-down interventions	Vertical program delivery and perception of community as beneficiaries, rather than equal stakeholders to vaccination program.
▪ Communication and capacity building interventions	Inter Personal Communication (IPC), Information Education and Communication (IEC), Behavior Change Communication (BCC), mass-media messaging for sensitization and awareness and capacity building trainings of workers, peer educators, health care workers and local stakeholders.
▪ Supply chain management	Ensuring supply of vaccines from the center to the ground levels.
(v) Transactional interventions	Dialogue with the community to build faith, trust and credibility for policymaker's/vaccine delivering system.
(vi) Vaccination delivery by frontline HCWs	Vaccination delivery by frontline healthcare workers (HCW) to vaccine eligible population.
Evolution of definition of Community Engagement	
2a. Comparing	Comparing different vaccine delivery/CE strategies across time or different vaccines, or comparing vaccination with other programs, urban and rural differences in delivery strategies and how things were/are done in other countries.
2b. Evidence base scientific approach	Learning from one vaccine experience, presence or lack of evidence/data/indicators of CE in the vaccine arena.
2c. Institutional strategic orientation	Organizational learnings and strategic orientations and shifts over time, across different vaccines and on the issue of community engagement in the vaccine space.
2d. Opinion based	Ideal opinions, more of rhetoric questions if that would work.
2e. Personal narrative	How his/her own conceptualization/role to engage with communities has changed over the years, in this and earlier organizations.
2f. Transformative	Macro transformations based on the burden of disease, vaccine availability like progressive state (HPV in Punjab) etc. Policy makers' thoughts to initiate new methods/models/strategies.
Support to promote Community Engagement	
3a. Global stakeholders	Support/help/solidarity of global partners and donors.
3b. Ideal or evidence of other regions or institutions	Support/help/solidarity of global partners and donors, or international collaborations of technical think tanks.

3c. National stakeholders	Support/help/partnership/solidarity of national level organizations and national level vaccine ambassadors or advocates. Political will of the Ministry/Prime Minister/Chief Minister.
3d. Respondent's own organization	Acknowledging enabling environment in the organization and support of the organizational head.
3e. Self	
(i) Duty	Performing his/her duty.
(ii) Responsibility	Self-motivated and performs beyond stipulated duty for innovative CE strategies.
Resources for Community Engagement in the Organization	
4a. Financial resources	Monetary resources within the organization which they have, or lack of finances.
4b. Human Resources	Human resources within the organization, e.g. Community outreach capacity of the organization - which they have or lack and should ideally possess.
Partnerships for Community Engagement	
5a. With global entities	Partnership with global organizations/and donors.
5b. With national organizations/technical bodies	Partnership with local organizations/technical body of the ministry/higher education institutions.
5c. With local CBOs, CSOs, Youth Clubs, communities etc.	Partnership with local CBOs, CSOs, Youth Clubs, communities etc.
5d. Ideal partnerships at organizational level	Ideal partnerships which would be helpful.
5e. Organizational	Existing partnerships of the organization/institution.
Community Level Enablers for Community Engagement	
6a. Local level influencers	Religious leaders, clubs, women's groups who influence positive vaccination decision among communities.
6b. Disease outbreaks	Certain outbreaks which compelled parents vaccinating their children.
Community Level Barriers for Community Engagement	
7a. Demand side barriers	
(i) Access	Child sick or travelling or nobody at home to take the child to the vaccination site.
(ii) Blackmailing and strategizing	Communities strategizing with Govt due to lack of trust – like ‘build the road then we will take the vaccines’ or ‘what must be the plot of the Govt. to vaccinate’ etc.
(iii) Fear and reluctance	Fear of illness or of death of child, fear of religious incompetence due to vaccination, arising from experience with this child, another child, neighbor’s child or media reports.
(iv) Lack of information	No knowledge about vaccines, no knowledge of vaccine schedules and lack of information about importance of the vaccines
(v) Myths and Rumors	Spreading of mis-information
(vi) Power structures in the society	Structural barriers related to class, caste, and gender, rural versus urban hindering reach of marginalized communities to getting vaccinated.
7b. Supply side barriers	
(i) Access	Health care worker not present in the session site, vaccines not there, vaccines there but have crossed the due date, vaccines could not reach the under-reached/under-served areas.
(ii) Power structures in the society	Structural barriers like power relations between researcher and community in the society, hindering reach of marginalized communities to getting vaccinated.
